# Thresholds for perception of direction of linear acceleration as a possible evaluation of the otolith function

**DOI:** 10.1186/1472-6815-5-5

**Published:** 2005-06-22

**Authors:** H Kingma

**Affiliations:** 1 University Hospital Maastricht, The Netherlands

## Abstract

**Background:**

Previous attempts to measure otolith function using ocular counter-rolling have shown poor sensitivity and specificity, thereby hindering a useful clinical application. We have conducted a study to investigate whether thresholds for the perception of the direction of linear acceleration might be an alternative for the clinical evaluation of otolith or statolith function.

**Methods:**

Perception of the direction of motion was evaluated in 28 healthy subjects while all external auditory and visual cues were eliminated. Whole body motion stimulus was generated by a motor driven linear sled at a stimulus frequency of 1 Hz at a linear acceleration ranging from 0 to maximum 40 cm/ s^2^. Subjects were required to correctly indicate the direction of motion (anterior-posterior or lateral) or whether they were stationary. Both velocity and acceleration thresholds were measured.

**Results:**

The median acceleration thresholds for the perception of direction of linear movement for anterior-posterior movement was 8.5 cm/s^2 ^and for lateral movement 6.5 cm/s^2^. According to the literature, acceleration thresholds depend on the stimulus profile whereas velocity thresholds do not. The median velocity thresholds for the perception of direction of linear movement for anterior-posterior movement was 13.5 cm/s and for lateral movement was 10.4 cm/s. The median velocity thresholds for the perception of direction of linear movement for anterior-posterior movement increased linearly with age, whereas the median velocity threshold for lateral movement was not correlated with age.

**Conclusion:**

The thresholds found in this study are lower than reported in the literature before which may be due to the repetative predictive sinusoidal stimulus which makes it relatively easy to lower the threshold by learning already within one test prophile.

The variablity is large in line with the previous literature, but our experiments indicate that variability decreases after a training session. We interprete the literature and our current results that linear velocity thresholds after some training might reflect the sensitivity of the otolith system per se.

## Background

The vestibular system is the sensory mechanism of the inner ear (labyrinth) that helps the body maintain its postural equilibrium. There are two distinct sets of end organs in the labyrinth: the utricle and saccule within the vestibule, which respond to linear accelerations and changes in the position of the head with respect to gravity; and the semicircular canals, which respond to rotational movements (angular acceleration). The information that these organs deliver is proprioceptive in nature. The left and right utricular sensory epithelia (maculae) are in the same, approximately horizontal plane and because of this position they appear to be the dominant partner and are more useful than the saccular maculae in providing information about the position of the head and its side-to-side tilts when a person is in an upright position. The maculae are stimulated by shearing forces between the otolithic membrane and the cilia of the hair cells beneath it. However, the measurement or quantification of this 'otolith function' in patients is very difficult.

Many methods have been proposed to try and evaluate otolith function: ocular counter-rolling (OCR) induced by lateroflexion, whole body roll, eccentric rotation and translational acceleration have all been explored and promoted as indicators of vestibular otolith function [1]. However, these methods showed poor sensitivity and specificity, thereby preventing a sound clinical application [1].

Lateroflexion or body roll is a simple physiological test that changes the orientation of the head and the otolith system in space and measures the responses, such as eye movements in response to a counter roll. The subject tilts their head to one side and, as a consequence the eyes counter roll to a certain extent. Unfortunately, this simple test is associated with very low sensitivity and specificity and there is a large overlap between patients and healthy subjects. For example, an extensive study in healthy subjects can reveal a wide range of ocular counter rolling, from 3 to 11 degrees, induced by this simple lateroflexion test [2]

It is also possible to measure responses to a linear acceleration (translation), which is also one of the specific stimuli for the otolith system. A linear sled device can change a subject's position in space, is motor driven and can move very fast (up to 1.2 G). However, if the sled is moving very fast and thus causing substantial motion, it is important to reduce the movements of the head using a mask specifically designed for each subject. In addition, the sled involves complex and advanced technology, and so is very expensive, and again there is limited sensitivity and a large overlap between patients and healthy subjects.

Responses can also be elicited by eccentric rotation in a 'human centrifuge' that can rotate up to 7 cycles/second and induce up to 6G [3]. Ocular counter rolling can be measured in this way, at constant rotation velocities with the amplitude of the response depending on the centrifugal force acting upon both labyrinths. When combining a centrifuge with a motor driven linear sled, it is also possible to test each of the labyrinths separately by rotation around one labyrinth in order to centrifuge the other labyrinth alone. Although this centrifuge technology has been used for many years, it is also associated with many problems and low sensitivities. The equipment is expensive, the eye movement responses are very small and correct position of the labyrinths difficult and responsible for false positive outcomes.

The aim of this study was therefore to investigate whether the thresholds for the perception of linear acceleration might allow for a better measure for the clinical evaluation of the otolith function than measurement of eye movements. When auditory and visual cues are excluded and body movement is minimised, the detection of dynamic motion stimuli of small intensity appears to be primarily dependent on the otolith and the somatosensory systems response to pressure changes on the body surface [4]. Previous studies have shown that when oscillatory stimuli of 0.3 – 0.4 Hz are employed, the thresholds for detection of linear movements in the horizontal plane range from 1.8 – 6.3 cm/s^2 ^for anterior-posterior (AP) accelerations and 1.9 – 5.7 cm/s^2 ^for lateral accelerations [5].

However, the literature shows that the acceleration thresholds vary with the stimulus profile used to determine the thresholds (sinus, parabolic, linear, steps), but that thresholds expressed in terms of velocity are more constant and less variable with the stimulus profile [6-9]. For example, Gianna et al observed mean normal thresholds of 4.84 cm/s2 using acceleration steps, 12.1 cm/s2 for linear ramps and 16.7 cm/s2 for parabolic stimuli [8,9]. Expressed in terms of velocity all thresholds were close to 20 cm/s. A practical problem of these stimulus profiles is that they require a long sled and that after each stimulus a deceleration period and adaptation period is required, which makes the test procedure long lasting. We therefore investigated the threshold for perception of the direction of linear horizontal motion using a raised cosine bell profile.

## Methods

Whole body motion stimulus was generated by a motor driven linear sled running on a horizontal track of 4.2 metres (maximum velocity 3.7 m/s; maximum acceleration 1.2 m/s^2 ^adjustable in steps of 1 cm/s^2^). The seat could be changed into one of two positions in which the AP or the traverse axis of the head was parallel to the direction of motion.

Twenty-eight healthy individuals with no previous complaints of dizziness and no history of audio-vestibular disease volunteered to participate in the study; 15 males and 13 females (22–60 years; seven subjects/decade). The subjects were seated upright with their feet on a footrest; head fixed against a headrest and the body restrained with safety belts. To eliminate external visual cues subjects were tested with eyes closed and in complete darkness; to eliminate auditory cues, subjects wore headphones; to mask proprioceptive cues, the sled was vibrated continuously by adding a sinusoidal signal to sled motor control profile (70 Hz sinus, 0.1 cm/s^2 ^peak amplitude). Upon request, all subjects indicated that this continuous vibration prevented them from using the motor vibrations as a cue for detection of sled motion per se.

The stimulus was a simple raised cosinus bell cycle of 0.1 Hz (5 periods maximum per test), with peak accelerations of 0 – 20 cm/s^2^. The impact of wind on movement perception is very difficult to prevent. However, speeds and accelerations were low, and the continuous cosinusoidal movement so smooth, that in a previous pilot study none of the subjects upon request indicated that they could make use of wind as a movement direction cue.

Preliminary data from patients with complete congenital bilateral vestibular areflexia (no responses to caloric irrigations and rotations, no Vestibular Evoked Myogenic Potentials, no galvanic induced body sway) showed velocity thresholds that exceeded the normal values observed in this study (>40 cm/s). This was considered to be a strong indication that proprioceptive cues played a minor role in perception of the sled movement in the experimental setup (results to be published elsewhere).

**Figure 1:**
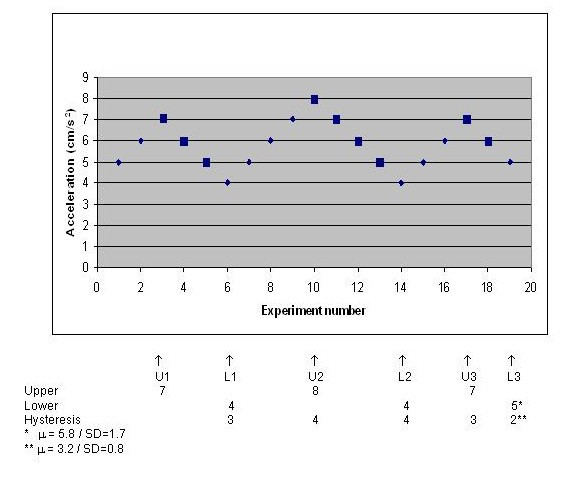
Schedule for threshold detection.

Thresholds for the perception of motion were obtained under two conditions: the seat into a position in which the AP axis of the head was parallel to the direction of motion and with the seat into a position in which the traverse axis of the head was parallel to the direction of motion. Subjects were required to correctly indicate whether they were moving forward or backward in an AP motion, left or right in lateral motion, or were stationary for all five outward and return cycles. Sled movement was initially set with a maximum acceleration of 5 cm/s^2 ^and the subjects were asked to indicate whether they could detect motion. Patient were not pre-informed about the possible sled profile. If they were unable to detect the direction of the sled correctly, the maximum acceleration was increased by 1 cm/s^2 ^until perception was correct. If detection was correct, acceleration was decreased by 1 cm/s^2 ^until detection failed. This procedure was repeated until six thresholds were detected (three upper levels and three lower levels) (Figure 1). In Figure 1, the large filled squares indicate when motion was detected, after which time acceleration was decreased by 1 cm/s^2 ^until the subject could not accurately determine motion or the direction of motion.

To rule out the possibility that differences in thresholds in the two directions might be attributable to training or to loss of concentration, back and forth movement was randomly performed as the first or last experiment.

The subjects participated in the study after signing their informed consents approved by the institutional Ethics Committee. All studies were conducted in accordance with the Declaration of Helsinki.

## Results

The Shapiro-Wilk test shows that both the thresholds in the AP motion and those in lateral motion are not normally distributed. Table 1 shows an overview of the acceleration and velocity threshold data. The AP and lateral thresholds were not significantly different (signed rank test, p < 0.192) and were correlated (Pearson; r = 0.561; p < 0.01) and the reproducibility was good (within 17%).

**Figure 2:**
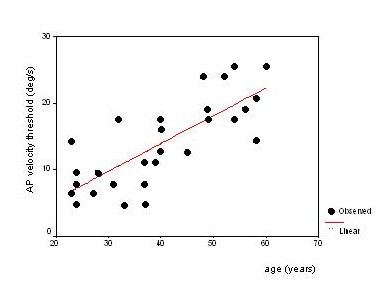
Velocity threshold of anterior-posterior linear movement as a function of age. Thresholds increase with age (median velocity threshold = -2.8 + 0.42 × age (p < 0.001, r = 0.775).

**Figure 3:**
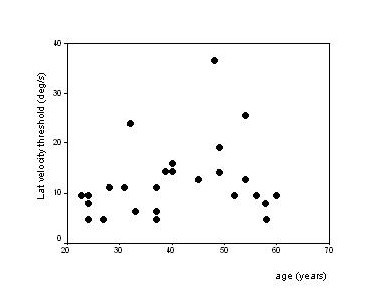
Velocity threshold of lateral linear movement as a function of age. The threshold for lateral movement is not correlated with age (p = 0.215, r = 0.242).

No significant correlation was found between thresholds and sex. Figures 2 and 3 show the thresholds as a function of age. The AP thresholds increased with age (median velocity threshold = -2.8 + 0.42 × age (p < 0.001, r = 0.775), but the threshold for lateral movement was not correlated with age (p = 0.215, r = 0.242).

## Discussion

The thresholds for perception of the direction of linear acceleration using an oscillation (cosine) observed in this study (median 6.5 – 8.5 cm/s^2^) were higher than those previously reported by Gundry [4]: 1.8 – 6.3 cm/s^2 ^for AP accelerations and 1.9 – 5.7 cm/s^2 ^for lateral accelerations. In addition, Gundry's results were obtained using oscillatory stimuli, with different techniques and equipment, and at frequencies of 0.3 – 0.4 Hz compared to 0.1 Hz used in our study [5]. These findings are also in agreement with those of Benson et al., who suggested that the reason thresholds for the detection of discrete movements in the AP direction decreased with stimulus frequency might be due to an increased contribution of propriocepsis to detection of movement at high frequencies [7]. Furthermore, in the studies performed by Gundry, only the threshold for perception of movement was evaluated and not that of movement direction [5]. In the current study, we detected a more specific threshold, in this case the threshold for detection of motion direction.

A major factor, however, is that acceleration thresholds reported in the literature vary widely with the stimulus profile used. It was suggested by Gianna et al. that velocity thresholds depend less upon the profile applied [8,9]. In the present study, median velocity thresholds ranging from 3 to 36.6 cm/s were measured. Mean thresholds were 13.9 cm/s for AP and 10.4 cm/s for lateral movements. In the literature, the mean velocity thresholds for *lateral *movements reported are all close to 20–22 cm/s (range 5 – 50 cm/s), which is substantially larger than the mean values observed in our study (AP: 13.5 cm/s and lateral: 12.2 cm/s; Table 1). The procedure (scoring criteria) followed by Gianna et al. [8,9] and Benson et al. [7] to determine velocity thresholds were different from the method used here and could account for differences. The oscillatory stimulus by its repetitive and predictive character makes it relatively easy to lower the threshold by learning already within one test [10].

There is also an age dependency that to our knowledge has not been reported previously in the literature: especially in elder people the velocity threshold for AP movements increases. The correlation with age is in agreement with the findings of Igarashi et al., who found that statoconia volume in elderly people was significantly less than that in young children [11]. It is not evident why this age dependency holds for AP and not for lateral movements. A fundamental aspect of importance might be that subjects in general are more habituated to low frequency long-lasting AP linear movements (trains, cars) than to lateral movements. Nevertheless, the thresholds for the two movement directions are not significant different and we do not see how habituation would affect age dependency.

It is well known that the vestibular system detects accelerations but controls eye velocity. When bode plots are made of the VOR it is obvious that within the normal sensitivity frequency range of the canals (roughly 0.1 – 10 Hz) the vestibular system detects head velocity and controls eye velocity (zero phase shift and constant gain). The observation that velocity threshold reflects vestibular sensitivity more robustly than acceleration thresholds complies with the assumption that the otolith system also detects primarily head velocity (via integration of the acceleration) and that motion perception is related to velocity, providing that sufficient acceleration occurs to stimulate the accelerometer (hair cells) in the labyrinth.

## Conclusion

The perception thresholds for linear acceleration might reflect otolith function better than the rudimentary, non-functional ocular counter rolling reflex but, similar to the OCR, a large range of normal values was observed. Our recent experiments indicate that this variability is primarily associated with the fact that many subjects are unfamiliar with the perception of minor linear accelerations. After a training session, variability seems to decrease; perception thresholds might then reflect more the sensitivity of the otolith system per se.

## Competing interests

The author(s) declare that they have no competing interests.

## Pre-publication history

The pre-publication history for this paper can be accessed here:



## Figures and Tables

**Table 1:** Threshold perception for AP and lateral accelerations

	**Median Threshold**	**Mean**	**Range**	**SD**
AP accelerations (cm/s^2^)	8.5	8.8	3 – 16	4.1
AP velocities (cm/s)	13.9	13.5	4.8 – 25.5	6.5

Lateral accelerations (cm/s^2^)	6.5	7.7	3 – 23	4.5
Lateral velocities (cm/s)	10.4	12.2	4.8 – 36.6	7.1
